# Environmental DNA sequencing dataset from Lake Erie algal blooms using Oxford Nanopore MinION

**DOI:** 10.1016/j.dib.2022.108688

**Published:** 2022-10-21

**Authors:** Alexander F. Koeppel, William J. Goodrum, Morgan M. Steffen, Louie L. Wurch, Stephen D. Turner

**Affiliations:** a Signature Science, LLC: 8329 N. Mopac Expressway, Austin, TX 78759, United States of America; b Elder Research: 300 W Main St STE 301, Charlottesville, VA 22903, United States of America; c James Madison University: Bioscience Building, MSC 7801, 951 Carrier Drive, Room 2001, Harrisonburg, Virginia 22807, United States of America

**Keywords:** MinION, Nanopore, eDNA, Harmful algal blooms, Freshwater ecology

## Abstract

Here we describe a publicly available environmental DNA (eDNA) sequence dataset, consisting of samples collected from a National Oceanic and Atmospheric Administration (NOAA) Great Lakes Environmental Research Laboratory (GLERL) on Lake Erie. We sequenced samples drawn from before, during, and after a 2019 *Microcystis* harmful algal bloom (HAB) using 3rd generation sequencing with the Oxford Nanopore MinION device. We classified the eDNA reads taxonomically, and estimated the abundances of all taxa in each sample. While the taxonomic data showed evidence of significant human and *E. coli* contamination, we found abundant *Mycrocystis*, especially in the samples drawn from bloom environments. The raw sequence data are available in the Sequence Read Archive (SRA) under accession number PRJNA812770. HABs pose a significant and increasing risk, both to human health and to the Blue Economy, and genomic approaches to early detection promise to help mitigate these risks. As such, this dataset could be of interest to freshwater ecology research teams, or any stakeholders interested in the detection and mitigation of HABs.

**Specifications Table**

**Table:** 

Subject	Environmental Genomics and Metagenomics
Specific subject area	Metagenomic analysis of freshwater microbes associated with harmful algal blooms
Type of data	Raw Metagenomic Data Metadata Table Figure
How the data were acquired	Freshwater eDNA samples collected from Lake Erie GLERL
Data format	Raw data (fastq.gz.file)
Description of data collection	DNeasy PowerWater^Ⓡ^ isolation kit was used to extract DNA from the water samples. DNA was then sequenced using the Oxford Nanopore MinION^Ⓡ^ device.
Data source location	Lake Erie NOAA GLERL stations WE13 and WE02. Latitude (approx.) 42.07 N Longitude (approx.) 81.34 W.
Data accessibility	We have deposited the raw sequences in the NCBI Sequence Read Archive (SRA) under accession number PRJNA812770, accessible at https://www.ncbi.nlm.nih.gov/bioproject/PRJNA812770/.

## Value of the Data

• The data provide a snapshot of the microbiota present before and during a *Microcystis* bloom.
• Harmful algal blooms, including *Microcystis* cause significant harm to human health and local economies.
• Understanding the taxonomic makeup of the freshwater microbiome can provide insights into the dynamics of bloom formation.
• Any stakeholders interested in the dynamics of HABs in general, and *Microcystis* blooms specifically may find these data to be of value.

## 1 Data Description

This dataset consists of raw environmental DNA (eDNA) reads from before and during a *Microcystis* bloom. We sequenced the DNA using the ONT MinION, and classified the reads taxonomically using the ONT What's In My Pot (WIMP) pipeline [1]. The complete dataset (all reads passing MinION QC) numbered 1,607,129 reads. Of these, 975,123 were successfully taxonomically classified. The dataset includes 10 samples (four drawn from bloom conditions, and six pre-bloom), with half of the samples in each bloom condition drawn from each of two different Lake Erie GLERL stations (WE02 and WE13) during the same bloom year (2019). Table 1 provides a subset of the metadata associated with these samples, including the GLERL from which the sample was drawn, the date of sequencing, bloom condition at the time of sampling, and DNA concentration. Because we sequenced some samples as a single run, and others as part of a multiplexed run, the multiplexing information is also included. Note that the DNA inputs for all runs was lower than called for by the MinION library preparation protocol (400ng of DNA), resulting in fewer reads than expected for an analysis of this type. Our sample biomass was low and concentrating the samples would have conflicted with our project goal of developing a field-portable HAB sequencing pipeline as described elsewhere [2]. As a result, read counts for our pre-bloom samples were only ∼2-fold higher on average than those of the reagent blanks. This reduction in throughput may have contributed to the unusual taxonomic results described below. The complete metadata for all samples is available on the SRA BioProject page.

**Table 1:** Subset of the available metadata about samples sequenced in this study, available at SRA accession number PRJNA812770.

Sample	Run date	GLERL	Condition	Input (ng/uL)	Run type	Reads (passing QC)
MP1_WE02_B1	2022-01-26	WE02	Bloom	22.9	Multiplexed	165800
MP1_WE13_B1	2022-01-26	WE13	Bloom	12.4	Multiplexed	131684
MP2_WE02_B2	2022-02-16	WE02	Bloom	6.16	Multiplexed	133788
MP2_WE13_B2	2022-02-16	WE13	Bloom	10.8	Multiplexed	164230
MP2_NC1	2022-02-16	NA	Negative control	LOW	Multiplexed	10156
FR1_WE13_PB6	2021-10-07	WE13	Pre-bloom	7.98	Single sample	609113
FR2_WE02_PB2	2021-11-08	WE02	Pre-bloom	0.353	Single sample	332514
MP1_WE02_PB1	2022-01-26	WE02	Pre-bloom	0.264	Multiplexed	10051
MP1_WE13_PB1	2022-01-26	WE13	Pre-bloom	0.053	Multiplexed	13004
MP2_WE02_PB2	2022-02-16	WE02	Pre-bloom	0.159	Multiplexed	12447
MP2_WE13_PB2	2022-02-16	WE13	Pre-bloom	LOW	Multiplexed	12221
MP1_RB1	2022-01-26	NA	Reagent blank	LOW	Multiplexed	5568
MP2_RB1	2022-02-16	NA	Reagent blank	LOW	Multiplexed	6553

**Fig. 1:**
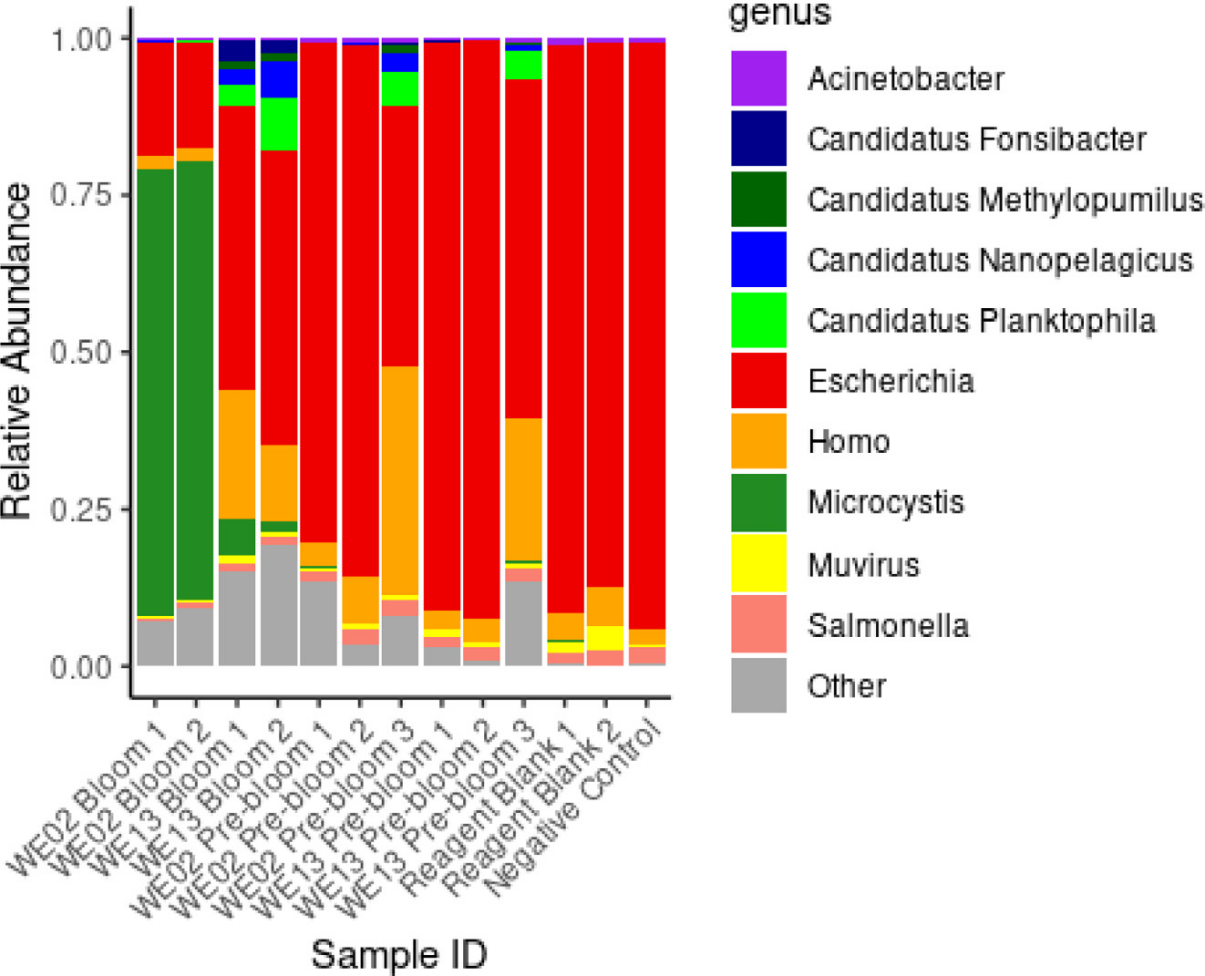
Relative taxon abundance (genus level) for all samples in the dataset. Top 10 most abundant genera across all samples are displayed. All other genera were classed as ‘Other.’ Sample names are the GLERL from which the sample was drawn, the condition (Bloom or Pre-bloom) and the replicate number. Reagent blank and negative control results are also included (right 3 columns). Note the presence of Escherichia and Homo classified reads (putative contamination) across all samples.

The results of the taxonomic classification are presented in Fig. 1. There was notable human and *E. coli* contamination present in several samples that was not revealed until taxonomic classification. While we took steps to track down and eliminate the contamination for later samples, we were unable to conclusively discover the source. Based on the quantities observed in the negative controls and reagent blanks, these included at minimum, 1,400 to several thousand reads classifying as *E. coli*, and from 50 to several hundred reads classifying as *H. sapiens*, and hundreds more classifying to other taxa, including *Shigella, Acinetobacter*, and *Microcystis* (though never more than 10 reads in any of the blanks or negative control). In some of the bloom-drawn samples (those from GLERL WE13) the abundance of *E. coli* was greater than that of *Microcystis* despite the fact that these were *Microcystis* blooms. Note that the abundance of *Microcystis* in the WE13 samples appears lower than in WE02, though still noticeably higher than in any of the pre-bloom or control samples. This could be in part due to lower bloom severity at the WE13 bloom during that sampling year. Additionally, the putative contaminants showed higher relative abundance in the WE13 samples and could have had a skewing effect on the abundance results.

**Fig. 2:**
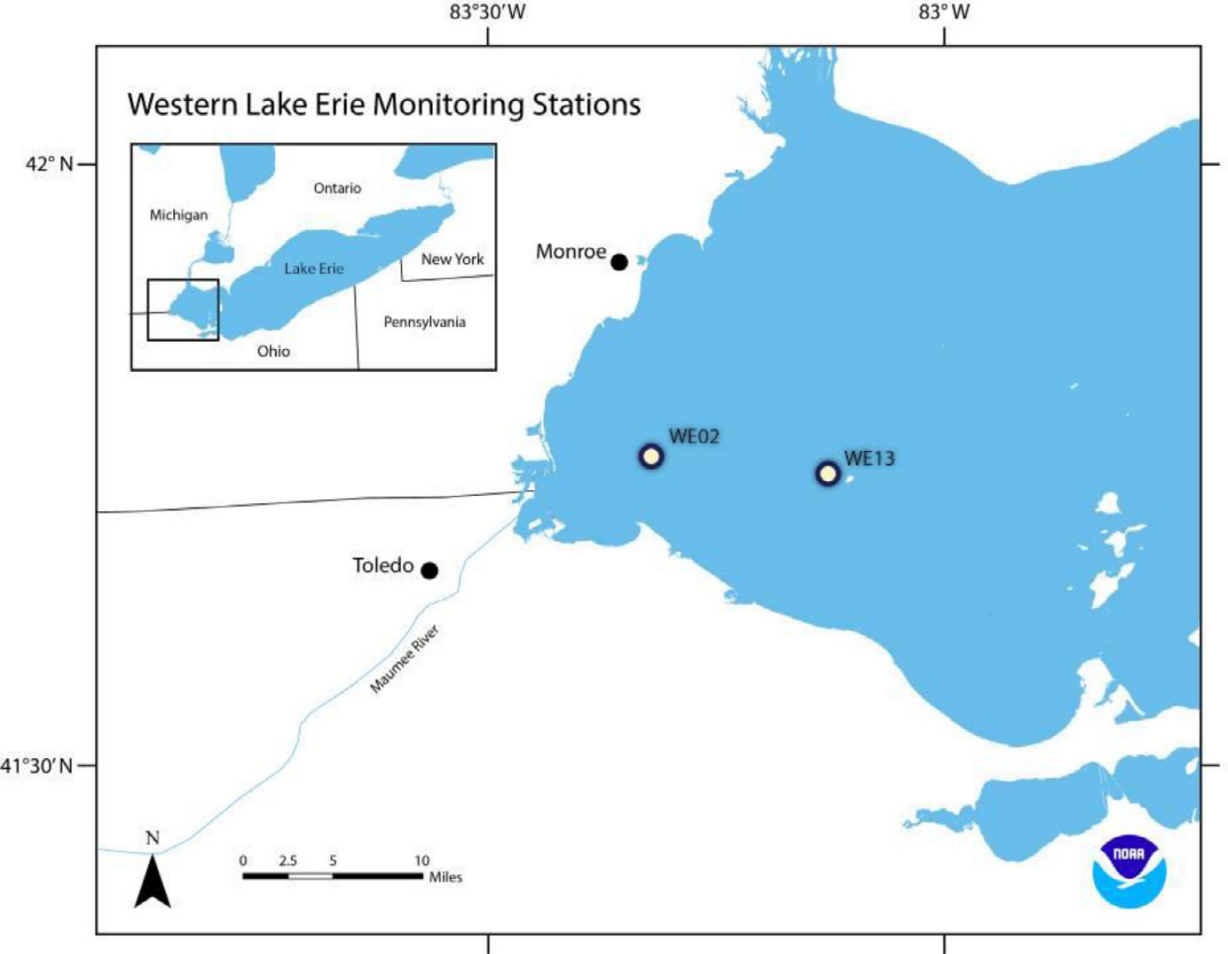
GLERL sites WE02 and WE13 from which water samples sequenced here were collected (adapted from https://www.glerl.noaa.gov/).

Collection sites are show in the map in Fig. 2 (adapted from www.glerl.noaa.gov). Details on the protocols for the collections at these sites are provided in the methods section.

## 2 Experimental Design, Materials and Methods

We sequenced water samples collected from NOAA GLERL stations WE02 and WE13 during a 2019 *Mycrocystis* bloom in Lake Erie. Sampling excursions from these NOAA GLERL stations in the 2019 bloom season took place during pre-, peak, and post-bloom conditions. The samples were drawn using standard collection protocols [3] at a water depth of 0.5m-1.5m. Metadata on the physical and chemical properties of the water at the time of sampling were also collected [4].

We collected surface water biomass onto 0.2 um Sterivex filters and kept on ice until they were returned to the lab where they were stored at -80C until extraction. We extracted the DNA from the samples, following a protocol adapted according to Cruaud *et al.* [5] We then prepared the libraries for MinION sequencing using Oxford Nanopore Technologies (ONT) sequencing kit (initially the Rapid Sequencing Kit (SQK-RAD004) but transitioning to the Rapid Barcoding Kit 96 (SQK-RBK110.96) for later runs). We estimated DNA concentrations for each sample using a Qbit analyzer. We sequenced the eDNA from those samples using the ONT MinION Mk1C device and performed base-calling and quality filtering using ONT's embedded MinKNOW software under the default settings. We then performed taxonomic classification using the ONT What's In My Pot (WIMP) pipeline. We computed relative abundance of taxa by dividing these genus-specific read counts by the total number of reads passing the QC filters for the same samples.

## Ethics Statements

AK and ST are employees of Signature Science, LLC. AK and WG were employees of Elder Research at the time this research were conducted. MS and LW are employees of James Madison University.

## CRediT authorship contribution statement

**Alexander F. Koeppel:** Conceptualization, Methodology, Software, Formal analysis, Investigation, Data curation, Writing – original draft, Writing – review & editing, Visualization, Funding acquisition. **William J. Goodrum:** Project administration, Funding acquisition. **Morgan M. Steffen:** Conceptualization, Methodology, Investigation, Writing – review & editing. **Louie L. Wurch:** Conceptualization, Methodology, Investigation, Writing – review & editing. **Stephen D. Turner:** Conceptualization, Methodology, Investigation, Data curation, Writing – review & editing, Supervision, Funding acquisition.

## Declaration of Competing Interest

The authors declare the following financial interests/personal relationships which may be considered as potential competing interests: Stephen Turner and Alex Koeppel are employees of Signature Science, LLC (SigSci). SigSci is a subawardee recipient of funding from the NOAA SBIR grant noted above, which supported a proof of concept study to establish the technical merit, feasibility, and commercial potential of a technology.

## Data Availability

Lake Erie eDNA HAB samples (Original data) (NCBI Sequence Read Archive).
